# Prevalence of Acetabular Dysplasia at a Mean age of 18 Years After Treatment for Neonatal hip Instability

**DOI:** 10.1097/BPO.0000000000002673

**Published:** 2024-04-16

**Authors:** Mari Maikku, Arja Marita Valkama, Pasi Ohtonen, Juhana Leppilahti

**Affiliations:** * Translational Medicine Research Unit, Medical Research Center Oulu, Oulu University Hospital, and University of Oulu, Oulu, Finland; † Department of Paediatrics, Oulu University Hospital, Wellbeing Services County of North Ostrobothnia, Pohde, Oulu, Finland; ‡ Research Unit of Clinical Medicine and Medical Research Center Oulu, Oulu University Hospital, and University of Oulu, Oulu, Finland; § Research Service Unit, Oulu University Hospital, Oulu, Finland

**Keywords:** neonatal hip instability, developmental dysplasia of the hip, hip dysplasia, long-term surveillance

## Abstract

**Background:**

The long-term radiological outcomes after the treatment of neonatal hip instability (NHI) in developmental dysplasia of the hip are unclear. Therefore, the prevalence of acetabular dysplasia at a mean age of 18 years after treatment was investigated. The relationship between acetabular dysplasia and hip discomfort has also been poorly established. Therefore, the differences in pain, hip-related quality of life (QOL), and hip impingement tests in hips with and without acetabular dysplasia were assessed.

**Methods:**

All 127 patients treated for NHI from 1995 to 2001 at the study hospital and meeting the inclusion criteria were invited to participate in this population-based follow-up. Of these individuals, 88 (69.3%) participated. The lateral center-edge angle (LCEA), Sharp’s angle (SA), and acetabular head index (AHI) were calculated for both hips from pelvic anterior–posterior radiographs. The Copenhagen Hip and Groin Outcome Score (HAGOS) questionnaire was completed for both hips separately; the total score, pain, and QOL subscores were calculated; and the impingement test was performed.

**Results:**

The prevalence of acetabular dysplasia, defined as an LCEA <20°, was 3.4%. Only the mean AHI (81.1%, SD 5.3) differed (-5.08, 95% CI -5.77 to -4.38, *P* <0.001) from previously described gender-specific and side-specific means, whereas the mean LCEA and SA did not. The odds ratio for a positive hip impingement test was 2.8 (95% CI: 1.11-7.05, *P* = 0.029) for hips with an LCEA <25° compared to hips with LCEA ≥25°. The hips with an SA ≥45° had a mean of 7.8 points lower for the HAGOS pain subscore (95% CI: 4.2-11.4, *P* <0.001) and a mean of 6.1 points lower for the HAGOS QOL subscore (95% CI: 2.1-10.2, *P* = 0.003) compared with hips with an SA <45°.

**Conclusions:**

The prevalence of acetabular dysplasia was low after treatment for NHI. Acetabular dysplasia seems to be as common in the general population as for those treated for NHI based on the LCEA. Only an SA ≥45° resulted in slightly more pain and lower hip-related QOL.

Neonatal hip instability (NHI) is the term used to describe when a newborn’s hip is dislocatable or dislocated in clinical tests or ultrasound examination within the first month of life as part of the very early phase of the disease spectrum of developmental dysplasia of the hip (DDH). Treated NHI is associated with a 2.6 times greater risk for total hip replacement in young adulthood.^1^ Unfortunately, long-term radiological results after treatment for NHI are still lacking.

When DDH treatment with an abduction device is started early, almost all patients have a normal radiological appearance of the hip in early childhood.^2–5^ However, the risk of persisting acetabular dysplasia after treatment is well known,^3,4,6,7^ and dysplasia might occur during growth.^8^ Studies with long follow-up periods have shown frequent acetabular dysplasia, but they have included large proportions of patients treated late.^3,8,9^ As late presentation is a risk factor for failure,^10^ previous studies’ results might not apply to patients who received treatment early.

The present study was the first population-based study to present long-term radiological results for treated NHI. This study investigated the prevalence of acetabular dysplasia, with Wiberg’s lateral center-edge angle (LCEA)^11^ as the primary outcome measure in 14- to 21-year-old adolescents treated for NHI. Primarily, it was hypothesized that there is a difference in the LCEA in participants treated for NHI compared with those of a previously reported cohort of adolescents in a normal population.^12^ Secondarily, it was hypothesized that there is a difference in Sharp’s acetabular angle (SA) and the acetabular head index (AHI) in participants treated for NHI compared with those of a previously reported cohort of adolescents in the normal population. Further, it was investigated if there was a difference in pain or hip-related quality of life (QOL) on the Copenhagen Hip and Groin Outcome Score (HAGOS)^13^ or impingement tests^14^ in hips with acetabular dysplasia. Previous studies have not evaluated hip-related QOL compared with hip morphology in those treated for DDH or NHI.

## METHODS

**FIGURE 1:**
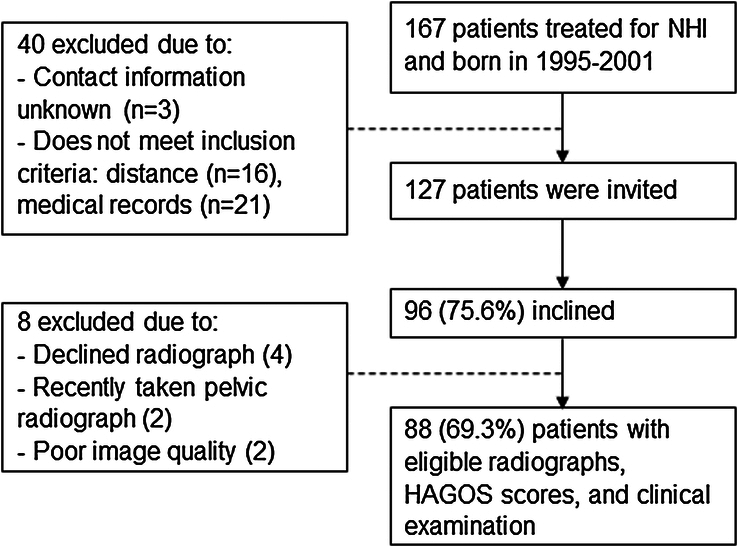
Flowchart of eligible radiographs and exclusion criteria for participants. HAGOS indicates Hip and Groin Outcome Score.

This was a long-term follow-up study of previous patients born from 1995 to 2001 who received early treatment (at <1 month of age) for NHI at Oulu University Hospital. The hospital medical records were searched for the relevant ICD-10 and ICD-9 codes. Patients whose treatment began after one month, with a relevant concomitant disease that could influence hip development, who had a preterm birth, with teratogenic luxation, or who were living more than 300 km from the hospital were excluded. The study was population-based as the hospital was the only institution providing treatment for DDH in the North Ostrobothnia district. Invitations to participate were sent to 127 previous patients who were a mean of 18 years old, and 88 (69.3%) had radiographs suitable for analysis (Fig. 1).

### Radiological Examination

The pelvis’s standard supine anterior–posterior radiograph (AP) was recorded at the pediatric radiology unit. Images with the greater trochanter seen laterally and the lesser trochanter partially superimposed on the femoral neck were accepted. The AP rotational image quality was ensured by visual inspection of the obturator rings’ symmetry, the iliac wings’ concavity, and acetabular teardrops. The Tönnis Foramen Obturator Index (FOI)^15^ was calculated, and images with FOI 0.7 to 1.8 were included for radiographic analysis.^16^

All image-based parameters were measured digitally by 1 author with HTML4/5-based software (neaLink, Neagen Ltd, Oulu, Finland). The author was proficient in the measurements by using measurements > 5 years in clinical practice. The most lateral point of the lateral rim was used in the measurements, and possible osteophytes were included. The inter-teardrop line was used as the transverse axis of the pelvis.^12^

### Outcome Measures

**FIGURE 2:**
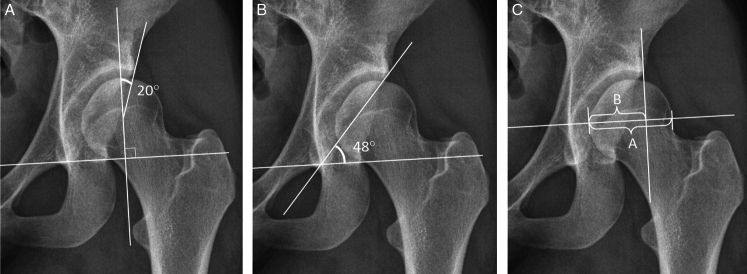
A radiograph of a borderline dysplastic hip shows the radiological parameters’ measurements: A. LCEA; B. SA; and C. AHI. AHI indicates acetabular head index; LCEA, lateral center-edge angle; SA, Sharp's acetabular angle.

The LCEA, the primary outcome measure, is the most common measurement for acetabular dysplasia. It is formed by a vertical line and a line connecting the femoral head center with the lateral edge of the acetabulum (Fig. 2a).^11^ An angle <20° was considered to be indicative of acetabular dysplasia.^12,17–19^ An additional threshold of ≤25° was included for borderline dysplasia.^20^

### Secondary Outcome Measures

The SA describes the acetabulum’s outlet angle formed between the acetabular roof’s lateral margin and the inferior aspect of the pelvic teardrop and inter-teardrop line (Fig. 2b).^21^ Values ≥45° were considered indicative of dysplasia.^17,22^

The AHI quantifies the horizontal coverage of the femoral head by the acetabulum (Fig. 2c). It is calculated by dividing the width of the femoral head beneath the acetabulum by the horizontal diameter of the femoral head.^23^ Values <75% are indicative of dysplasia.^12,17,24^

The participants were asked to complete the HAGOS for both hips separately.^13^ HAGOS is considered the most accurate questionnaire for evaluating hip pain in young adults, as it was developed for physically active patients.^25^ The questions were rated on a Likert scale of 5 items. The individual scores were converted to produce the total score, the pain, and the QOL subscale summary scores between 0 and 100, where 0 represented extreme hip and groin problems, and 100 represented no hip and groin problems.

Physical examinations were performed by Mari Maikku. The examiner was blinded to hip complaints, radiographs, and the side of the prior NHI. The protocol was standardized and has been described before in a previous study.^26^ The impingement test (flexion, abduction, and internal rotation) was performed, with groin pain indicating a positive test.^14^

### The Demographic Data and Medical Histories Were Recorded

This study complied with the ethical principles outlined in the Declaration of Helsinki. The study was registered in the National Clinical Trials Registry (NCT02431871). The local ethics committee accepted the protocol. All participants provided informed consent.

### Statistical Analysis

Summary measurements were presented as means with SDs unless otherwise stated. When using the results of both hips, a linear mixed model (LMM) or generalized linear mixed model (GLMM) was used for the analyses. The participant was set as a random effect in the LMM and GLMM due to dependent hips. When comparing with the Bergen Birth Cohort, participant and side (left/right) were set as random effects. The population-based 1989 Bergen Birth Cohort was a long-term follow-up of a large, randomized controlled trial undertaken at Bergen, Norway, designed to assess different ultrasound screening strategies for DDH. The follow-up focused on hip dysplasia.

The radiological measurements between affected and contralateral hips in unilateral NHI were compared using the paired samples *t*-test for continuous data and conditional logistic regression for binomial data. The mean differences with a 95% confidence interval (95% CI) were calculated for the LMM and t-test. Odds ratios (ORs) with a 95% CI were used as the result of the GLMM and conditional logistic regression.

Two-tailed *P*-values were reported, and SAS (version 9.4, SAS Institute Inc., Cary, NC, USA) and SPSS for Windows (IBM SPSS Statistics for Windows, version 28.0, 2016 release, IBM Corp., Armonk, NY, USA) were used for the analyses. The checklist of items for cohort studies (Strengthening the Reporting of Observational Studies in Epidemiology statement; http://www.strobe-statement.org) was followed when reporting the data.

## RESULTS

The mean age of the 88 participants (69 female, 19 male) was 18.2 years (SD 2.1, range 14–22). Neonatal treatment in von Rosen type splint (Coxa-splint, Pedihealth, Finland) had been started from a mean age of 0.54 weeks (SD 0.70). The mean treatment duration was 8.8 weeks (SD 2.6). There were altogether 118 hips with NHI, including 41 hips positive for the Ortolani test and 56 hips positive for the Barlow test. In 21 hips in 15 patients, the treatment was started based on clinical instability at the maternity ward, and an additional ultrasound examination was performed at a mean of 1.3 weeks of age (SD 1.1). Of those, 11 hips were Barlow positive, 4 were Ortolani positive, and 6 had suspicious findings but no apparent instability at the maternity ward.

In 1 patient, treatment failed, resulting in a spica cast treatment. After treatment, the patients had routine follow-ups at a mean age of 5.9 months (SD 0.63) and 13.5 months (SD 2.2). The surveillance ended at the second follow-up in 75 patients (85.2%). Surveillance was transferred to another hospital for three participants. Among 11 participants, the follow-ups continued at the study hospital until a mean age of 1.9 years (SD 0.95, range 1.2 to 4.2). Of those, 4 individuals still had slight acetabular dysplasia or lateralization of the femoral head present in 7 hips. One had a suspicion of avascular necrosis of the femoral head. No one had required a later surgical procedure.

The prevalence of an LCEA <20° was 3.4% (3 patients, all unilaterally). All 3 hips with an LCEA <20° also had an SA ≥45° and an overall dysplastic appearance in the radiograph taken for this study, but they all had a normal acetabular index (AI) and AHI at 1 year of age. The prevalence of an SA ≥45° and AHI <75% were 19.3% and 14.8%, respectively.

Of all 176 hips in 88 participants, 149 (84.7%) had no radiographic findings indicating dysplasia; however, 19 hips (10.8%) had one measurement showing dysplasia, 6 hips (3.4%) had 2 measurements, and only 2 (1.1%) had all 3 measurements indicating dysplasia. The proportions of hips with borderline dysplasia or dysplasia are presented in Table 1.

**TABLE 1:** Proportions of Hips with Measures Indicating Borderline Dysplasia or Dysplasia in Both Hips by Sex and Hip Side

	All hips	Gender		Hip side	
Variable	(N=176)	Female hips (N=138)	Male hips (N=38)	Right hips (N=88)	Left hips (N=88)
LCEA < 25°, n (%)	26 (14.8)	22 (15.9)	4 (10.5)	10 (11.4)	16 (18.2)
SA ≥ 45°, n (%)	19 (10.8)	17 (12.5)	2 (5.3)	9 (10.2)	10 (11.4)
AHI < 75%, n (%)	15 (8.5)	13 (9.4)	2 (5.3)	5 (5.7)	10 (11.4)

AHI indicates acetabular head index; LCEA, lateral center-edge angle; SA, Sharp’s acetabular angle.

The mean LCEA and SA did not differ significantly from sex- and side-specific means in the Bergen Birth Cohort (Table 2). However, the mean sex-adjusted and side-adjusted AHI was 5.08 lower for the present study (*P*<0.001).

**TABLE 2:** The Mean Values and SDs of Dysplasia Measurements in Comparison with the 17 to 20-year-old Population in the Bergen Birth Cohort Study. Comparisons were Made Considering Sex-specific and Side-specific Values

	Present study					Bergen Birth Cohort						
	All hips	Female		Male		Female		Male				
Variable	(N=176)	Right (N=138)	Left (N=138)	Right (N=19)	Left (N=19)	Right	Left	Right	Left	Diff.	p	95% CI
LCEA (°), mean (SD)	31.6 (5.8)	31.5 (5.8)	30.5 (5.9)	33.8 (4.8)	33.4 (5.8)	30.1 (6.1)	31.4 (6.0)	32.1 (6.1)	32.8 (5.8)	0.47	0.21	-0.27 to 1.21
SA (°), mean (SD)	40.6 (3.4)	41.0 (3.2)	41.1 (3.4)	39.7 (2.9)	38.3 (3.4)	40.7 (3.5)	40.8 (3.6)	38.8 (3.5)	38.7 (3.5)	0.30	0.18	-0.14 to 0.73
AHI (%), mean (SD)	81.1 (5.3)	81.1 (5.4)	80.5 (5.2)	81.8 (6.1)	83.0 (4.9)	85.6 (6.6)	86.8 (6.7)	85.6 (6.3)	86.9 (6.0)	-5.08	<0.001	-5.8 to -4.4

AHI indicates acetabular head index; Diff, difference in means; LCEA, lateral center-edge angle; SA, Sharp’s acetabular angle.

As dysplasia was rare, a small subgroup analysis was performed for clinical interest. There were 58 patients treated for unilateral NHI. The measurements of acetabular dysplasia did not differ significantly between affected and contralateral hips in unilateral NHI (Table 3). This was also true for Ortolani-positive hips and their contralateral stable hips.

**TABLE 3:** The Differences Between Affected and Contralateral Hips in all Unilateral NHI and Unilateral Ortolani-positive NHI

	Mean (SD)					
All unilateral NHI	Affected hips (N =58)	Contralateral hips (N=58)	Diff.	*p*	95% CI
LCEA (°), mean (SD)	31.5 (5.7)	31.7 (5.5)	-0.24	0.65	−1.27 to 0.80
SA (°), mean (SD)	40.2 (3.3)	40.6 (3.2)	-0.34	0.35	−1.06 to 0.38
AHI (%), mean (SD)	80.4 (5.0)	81.1 (4.8)	-0.64	0.20	−1.62 to 0.34
	Number (%)	Number (%)	OR	*p*	95% CI OR
LCEA < 25°, n (%)	9 (15.5)	6 (10.3)	2.50	0.45	0.41 to 26.25
SA ≥ 45°, n (%)	5 (8.6)	6 (10.3)	0.75	>0.90	0.11 to 4.43
AHI < 75%, n (%)	6 (10.3)	3 (5.2)	4.0	0.38	0.40 to 197
Ortolani-positive unilateral NHI	Affected hips (N =18)	Contralateral hips (N=18)	Diff.	*p*	95% CI
LCEA (°), mean (SD)	30.0 (3.6)	30.6 (4.1)	-0.58	0.55	−2.56 to 1.41
SA (°), mean (SD)	41.4 (3.1)	41.1 (3.2)	-0.25	0.66	−1.44 to 0.94
AHI (%), mean (SD)	80.0 (2.9)	81.5 (3.5)	-1.52	0.13	−3.57 to 0.53

AHI indicates acetabular head index; CI, Confidence interval; Diff, difference in means; LCEA, lateral center-edge angle; SA, Sharp’s acetabular angle; SD, Standard deviation

The mean HAGOS total score was lower for hips with an LCEA <25° with a mean difference of 2.8 (*P*=0.040, Table 4). The hips with an SA ≥45° had a significantly lower HAGOS pain subscore with a mean difference of 7.8 (*P*<0.001) and lower HAGOS QOL subscore with a mean difference of 6.1 (*P*=0.003). Additionally, the OR for the positive impingement test was 2.8 for hips with an LCEA <25° (*P*=0.029, Table 4).

**TABLE 4:** The Mean Differences with a 95% CI in an LMM for the HAGOS Total Score, Pain Subscore, and QOL Subscore, as well as the OR in a GLMM for Positive Impingement Tests and for Measurements Indicating Dysplastic and Nondysplastic Hips

	LCEA				SA				FHEI			
Variable	< 25° (N=24)	> 25° (N=148)	mean difference (95% CI)	*p*	≥ 45° (N=19)	< 45° (N=153)	mean difference (95% CI)	*P*	< 75% (N=14)	> 75% (N=158)	mean difference (95% CI)	*P*
HAGOS total (mean, SD)	88.2 (12.9)	92.7(7.9)	2.8 (0.1 to 5.5)	0.040	85.8(13.0)	92.9(7.9)	2.3 (-0.2 to 4.8)	0.076	90.9(9.1)	92.2(8.9)	1.8 (-1.3 to 4.8)	0.25
HAGOS pain (mean, SD)	91.5 (13.1)	94.9(6.7)	1.8 (-0.5 to 4.0)	0.13	87.5(13.4)	95.3(6.6)	7.8 (4.1 to 11.4)	<0.001	93.9(9.2)	94.5(7.9)	0.6 (-3.8 to 4.9)	0.81
HAGOS QOL (mean, SD)	87.9 (18.4)	93.2(10.3)	3.6 (-0.7 to 7.9)	0.096	83.4 (17.7)	93.6(10.4)	6.1 (2.1 to 10.2)	0.003	94.3(7.0)	92.3(12.1)	0.03 (-4.9 to 5.0)	>0.90
	OR		95% CI for OR	*P*	OR		95% CI for OR	*P*	OR		95% CI for OR	*P*
Impingent positive	2.8	1 (ref)	1.1 to 7.1	0.029	2.3	1 (ref)	0.81 to 6.5	0.12	1.3	1 (ref)	0.38 to 4.5	0.67

CI indicates confidence interval; FHEI, femoral head extrusion index; GLMM, generalized linear mixed model; HAGOS, The Copenhagen Hip Outcome Score; LCEA, Lateral center-edge angle; LMM, linear mixed model; OR, odds ratio; QOL, quality of life; ref, Reference group; SA, Sharp’s acetabular angle

## DISCUSSION

This population-based long-term follow-up study investigated the prevalence of acetabular dysplasia after the treatment of NHI with LCEA as the primary outcome measure. Only 3.4% of participants had an LCEA <20°. The sex-specific and side-specific mean values of LCEA did not show a significant difference between a previously described similar-aged cohort.^12^ Based on the LCEA, treated NHI did not predispose patients to acetabular dysplasia in 18 years of follow-up, and the primary hypothesis can be refuted. The prevalence of an SA ≥45° and AHI <75° were higher. The sex-specific and side-specific mean values of the AHI compared to previously described similar-aged cohort^12^ showed a significant difference. Partly, the secondary hypothesis can also be refuted.

Several studies have investigated the LCEA reference range based on the 2.5th percentile, and the lower limit of ~<20° is widely recognized.^12,17,19^ The 3.4% prevalence of an LCEA <20° in this study was similar to the Bergen Birth Cohort (3.3%)^22^ and a Danish population-based cohort (3.4%).^17^ Moreover, the present sex-specific and side-specific mean LCEA values are comparable to those for previous population-based European cohort studies.^17,27^ It has been speculated that dysplasia is more common in Asia than in Europe.^18^ The overall prevalence of LCEA <20° was 13.9%^18^ and 15.0%^28^ in cohorts of Japanese volunteers and asymptomatic South Korean adults, respectively. Also, Japanese adolescents had slightly lower mean values of LCEA (27.5°) than in the present study.^29^ Consequently, the present results of the low prevalence of acetabular dysplasia after NHI apply to Europe.

Previous studies have suggested a tendency in patients treated for DDH to develop dysplasia during growth.^4,7^ The two long-term follow-ups of DDH until skeletal maturity had participants treated from a mean age of 4 months and found acetabular dysplasia was common.^8,9^ The present results emphasize the excellent prognosis of NHI in contrast to DDH with later treatment. Also, in the subgroup analysis, even the hips positive for the Ortolani test did not have more dysplastic measures compared with the nonaffected hips in unilateral NHI.

The studies with follow-ups until school age in patients with early treatment of DDH support the present results. Patients with successful early treatment had a similar mean LCEA and SA to those in the present study at a mean age of 10.2 years.^5^ Cashman et al found a mean AI value 4° larger than the values of the normal population at a mean surveillance of 6.5 years, but the prevalence of an LCEA <20° was only 3.5%, similar to the findings in the present study.^4^

The high prevalence of an SA ≥45° and AHI<75° in the present study were interesting. Also, the mean AHI was significantly more dysplastic in the present study than in the Bergen Birth Cohort. Two recent Japanese studies reported a similar mean value for the AHI compared with that found in the present study.^18,29^ Nishimura et al reported an even higher mean SA and speculated whether the propensity for dysplastic values was caused by the young age (12 to 18 years) of the participants in their cohort.^29^ The clinical significance of these findings is unknown as the most important measurement for acetabular dysplasia, LCEA, did not indicate a higher rate of dysplasia.

In the present study, the mean difference for the HAGOS total score was only 2.8 points lower for hips with an LCEA ≤25° compared with hips with LCEA >25°, which does not meet clinical relevance. However, an LCEA ≤25° predicted a positive hip impingement test with an OR of 2.8. The hips with an SA ≥45° had a mean 7.8 points lower pain subscore and a mean 6.1 points lower QOL subscore compared with hips with an SA < 45°, which met the smallest detectable change value at the group level reported by the questionnaire developers.^13^ The reference ranges for the LCEA, SA, and AHI are based on an arbitrary SD of 2 or percentiles of 2.5 or 5, not clinical relevance. To our knowledge, this is the first study to report that a high SA could be associated with hip complaints in a cohort setting. Therefore, further investigation should determine whether the SA is more predictive of hip complaints than the LCEA. In a previous study, there was no significant difference in the HAGOS scores for patients treated for NHI and age-matched and sex-matched controls.^26^ There are few reports on the association of acetabular dysplasia with hip complaints in the general population. In Japanese older adults, a low LCEA was associated with hip pain, but only 1.3% of hips with an LCEA of 20°‬ to 25° had pain within the past month.^18^ Contrary to our findings, Engesaeter et al found no association between an LCEA <20°, SA ≥45°, or multiple radiological signs of hip dysplasia and The Western Ontario and McMaster Universities Osteoarthritis Index.^22^

### Strengths

The present study had some considerable strengths. First, the neonatal treatment was very similar in all participants. The setting was population-based as all the DDH patients in the district were treated at the same hospital, to which other hospitals referred suspected cases of DDH. The health care in Finland is based on a universal, publicly funded system that enables the centralized treatment of specific diseases. The participation rate (69.3%) was very high, especially considering the vast recruitment area and the extensive 18-year follow-up period. It was important to achieve high participation. If only those with complaints would participate, the prevalence of dysplasia could be false. The high rate was based on a few key things: (1) universal health care enables mainly up-to-date contact information, and the citizens are known to be “obedient.” (2) The participants were promised a voucher worth ~20 dollars as a small compensation for their effort. (3) We were persistent in contacting every patient with up to 3 letters and 2 phone calls, and patients received several options on how to contact us back. (4) The patients had the imaging and physical examinations on the same day, and the questionnaires were returned before that, so all returned the forms.

### Limitations

This study had limitations. The main limitation was the applicability of the present results to currently treated patients. Nearly all the participants had started treatment before 1 week old and based on clinical examination, and therefore, patients had the potential for spontaneous resolution. Barlow himself reported that as much as 88% of hips with positive Barlow test might stabilize in 2 months.^30^ The natural history of Ortolani-positive hips is less studied. However, the American Academy of Orthopaedic Surgeons Clinical Practice Guideline suggests immediate or delayed (2 to 9 weeks of age) treatment of Barlow and Ortolani-positive hips.^31^ Our institution’s standard care pathway for DDH endorses an ultrasound examination at the age of 2–3 weeks without treatment being started for all infants with NHI. Subsequently, some of the treated patients would not receive treatment anymore. The treatment rate at that time at our institution was 0.7%^32^ and is currently 0.29%.^33^ Thus, interpreting these results should be done with caution and cannot be extended to the long-term sequelae of today’s treatment for DDH patients. This might also influence the overall low rate of dysplasia.

Fifteen patients received additional neonatal ultrasounds before treatment at a very early age. Ultrasound before six weeks of age has been held unreliable.^34^ However, it should be noted that only 6 of 118 affected hips, including those treated after ultrasound, had negative Ortolani-Barlow test at the maternity ward.

Moreover, the Bergen Birth Cohort does not necessarily represent the young population in Finland. However, it had the same mean age of the participants and an excellent study protocol and reporting. Moreover, several studies have already demonstrated the reproducibility of LCEA, SA, and AHI.^17,18,29^ We did not control for femoro-acetabular impingement or other hip pathology in the analysis or lifestyle factors, such as smoking, which leaves the possibility of residual confounding. Finally, as acetabular dysplasia was uncommon, the sample size for evaluating the association between hip-related QOL and radiographic parameters was too small to draw definite conclusions.

In this population-based long-term follow-up, participants treated for NHI were not predisposed to acetabular dysplasia, defined by an LCEA <20°. The relationship between radiological hip dysplasia and hip discomfort should be further investigated in a large population-based cohort.

## CONCLUSIONS

Acetabular dysplasia was not more common in adolescents treated for NHI than in previous population-based cohorts.

## References

[1] Engesæter IØ Lie SA Lehmann TG . Neonatal hip instability and risk of total hip replacement in young adulthood. Acta Orthop. 2008;79:321–326. 18622834 10.1080/17453670710015201

[2] Dunn PM Evans RE Thearle MJ . Congenital dislocation of the hip: Early and late diagnosis and management compared. Arch Dis Child. 1985;60:407–414. 4015144 10.1136/adc.60.5.407 PMC1777324

[3] Tucci JJ Kumar SJ Guille JT . Late acetabular dysplasia following early successful Pavlik harness treatment of congenital dislocation of the hip. J Pediatr Orthop. 1991;11:502–505. 1860952 10.1097/01241398-199107000-00016

[4] Cashman JP Round J Taylor G . The natural history of developmental dysplasia of the hip after early supervised treatment in the Pavlik harness. A prospective, longitudinal follow-up. J Bone Joint Surg Br. 2002;84:418–425. 12002504 10.1302/0301-620x.84b3.12230

[5] Allington NJ . Successful Pavlik Harness treatment for developmental dysplasia of the hip and normal X-Ray at the age of 2 years: Is a longer follow-up necessary? J Pediatr Orthop. 2017;37:328–331. 26422392 10.1097/BPO.0000000000000657

[6] Dornacher D Lippacher S Reichel H . Mid-term results after ultrasound-monitored treatment of developmental dysplasia of the hips: To what extent can a physiological development be expected? J Pediatr Orthop B. 2013;22:30–35. 23014065 10.1097/BPB.0b013e32835957a1

[7] Wenger D Duppe H Tiderius CJ . Acetabular dysplasia at the age of 1 year in children with neonatal instability of the hip. Acta Orthop. 2013;84:483–488. 24171679 10.3109/17453674.2013.850009 PMC3822134

[8] Fujioka F Terayama K Sugimoto N . Long-term results of congenital dislocation of the hip treated with the Pavlik harness. J Pediatr Orthop. 1995;15:747–752. 8543603 10.1097/01241398-199511000-00006

[9] Nakamura J Kamegaya M Saisu T . Treatment for developmental dysplasia of the hip using the Pavlik harness: Long-term results. J Bone Joint Surg Br. 2007;89:230–235. 17322441 10.1302/0301-620X.89B2.18057

[10] Omeroglu H Kose N Akceylan A . Success of Pavlik Harness treatment decreases in patients >/= 4 months and in ultrasonographically dislocated hips in developmental dysplasia of the hip. Clin Orthop Relat Res. 2016;474:1146–1152. 26047647 10.1007/s11999-015-4388-5 PMC4814405

[11] Wiberg G . Studies on dysplastic acetabula and congenital subluxation of the hip with Special References to the Complication of Osteoarthritis. Acta Chirurgica Scandinavica. 1939;83:(suppl 58)5–135.

[12] Laborie LB Engesæter IØ Lehmann TG . Radiographic measurements of hip dysplasia at skeletal maturity--new reference intervals based on 2,038 19-year-old Norwegians. Skeletal Radiol. 2013;42:925–935. 23354528 10.1007/s00256-013-1574-y

[13] Thorborg K Holmich P Christensen R . The Copenhagen Hip and Groin Outcome Score (HAGOS): development and validation according to the COSMIN checklist. Br J Sports Med. 2011;45:478–491. 21478502 10.1136/bjsm.2010.080937

[14] Troelsen A Mechlenburg I Gelineck J . What is the role of clinical tests and ultrasound in acetabular labral tear diagnostics? Acta Orthop. 2009;80:314–318. 19421915 10.3109/17453670902988402 PMC2823204

[15] Tönnis D . Normal values of the hip joint for the evaluation of X-rays in children and adults. Clin Orthop Relat Res. 1976;119:39–47. 954321

[16] Jacobsen S Sonne-Holm S Lund B . Pelvic orientation and assessment of hip dysplasia in adults. Acta Orthop Scand. 2004;75:721–729. 15762262 10.1080/00016470410004094

[17] Jacobsen S Sonne-Holm S Søballe K . Hip dysplasia and osteoarthrosis. Acta Orthop. 2005;76:149–158. 16097538 10.1080/00016470510030517

[18] Iidaka T Muraki S Oka H . Radiographic measurements of the hip joint and their associations with hip pain in Japanese men and women: The Research on Osteoarthritis/osteoporosis Against Disability (ROAD) study. Osteoarthr. Cartil. 2017;25:2072–2079. 10.1016/j.joca.2017.08.008 28844568

[19] Werner CML Ramseier LE Ruckstuhl T . Normal values of Wiberg’s lateral center-edge angle and Lequesne’s acetabular index--a coxometric update. Skeletal Radiol. 2012;41:1273–1278. 22584462 10.1007/s00256-012-1420-7

[20] McClincy MP Wylie JD Kim YJ . Periacetabular osteotomy improves pain and function in patients with lateral center-edge angle between 18° and 25°, but are these hips really borderline dysplastic? Clin Orthop Relat Res. 2019;477:1145–1153. 30272611 10.1097/CORR.0000000000000516 PMC6494304

[21] Sharp IK . ACETABULAR DYSPLASIA. J Bone Joint Surg Br. 1961;43-B:268–272.

[22] Engesaeter IO Laborie LB Lehmann TG . Prevalence of radiographic findings associated with hip dysplasia in a population-based cohort of 2081 19-year-old Norwegians. Bone Joint J. 2013;95-B:279–285. 23365042 10.1302/0301-620X.95B2.30744

[23] Heyman CH Herndon CH . Legg-Perthes disease; a method for the measurement of the roentgenographic result. J Bone Joint Surg Am. 1950;32 A:767–778. 14784485

[24] Cooperman DR Wallensten R Stulberg SD . Acetabular dysplasia in the adult. Clin Orthop Relat Res. 1983;175:79–85. 6839611

[25] Thorborg K Tijssen M Habets B . Patient-Reported Outcome (PRO) questionnaires for young-aged to middle-aged adults with hip and groin disability: A systematic review of the clinimetric evidence. Br J Sports Med. 2015;49:812. 25586913 10.1136/bjsports-2014-094224

[26] Maikku M Ohtonen P Valkama M . Treatment outcome of neonatal hip instability. Bone Joint J. 2020;102-B:1767–1773. 33249896 10.1302/0301-620X.102B12.BJJ-2020-0266.R2

[27] Fischer CS Kühn JP Ittermann T . What Are the reference values and associated factors for center-edge angle and alpha angle? A population-based study. Clin Orthop Relat Res. 2018;476:2249–2259. 30024461 10.1097/CORR.0000000000000410 PMC6259987

[28] Kim CH Park JI Shin DJ . Prevalence of radiologic acetabular dysplasia in asymptomatic Asian volunteers. J Hip Preserv Surg. 2019;6:55–59. 31069096 10.1093/jhps/hnz001 PMC6501437

[29] Nishimura T Watanabe H Taki N . Standard radiographic values for the acetabulum in Japanese adolescents: A cross-sectional study. BMC Musculoskelet Disord. 2023;24:257. 37013503 10.1186/s12891-023-06368-z PMC10069018

[30] Barlow TG . Treatment of congenital dislocation of the hip in the newborn. Proc R Soc Med. 1963;56:804–806. 14080075 10.1177/003591576305600920 PMC1897214

[31] American Academy of Orthopaedic Surgeons Detection and Nonoperative Management of Pediatric Developmental Dysplasia of the Hip in Infants Up to Six Months of Age. Evidence-Based Clinical Practice Guideline. [AAOS Web site]. 2022. Accessed December 27, 2023. https://www.aaos.org/pddhcpg.

[32] Valkama AM, Aarnivala HI, Sato K, Harila V, Heikkinen T, Pirttiniemi P. Plagiocephaly after Neonatal Developmental Dysplasia of the Hip at School Age. *J Clin Med*. 2019;9. 10.3390/jcm9010021 PMC7019875 31861739

[33] Maikku M. Rantala T. Valkama M. The Ortolani test has a high positive predictive value in clinical hip screening for developmental dysplasia of the hip. Acta Paediatrica. 2023;112:1586–1591. 36928888 10.1111/apa.16757

[34] Chang WC Hsu KH Su YP . Association between early sonographic findings and acetabular index at the age of 6 months: A prospective observational study. BMC Pediatr. 2022;22:228. 35473514 10.1186/s12887-022-03268-4 PMC9040221

